# Assessing mutation-clinical correlations and treatment outcomes in Vietnamese non-small cell lung cancer patients

**DOI:** 10.1016/j.plabm.2025.e00477

**Published:** 2025-05-23

**Authors:** Hoang-Bac Nguyen, Bang-Suong Nguyen-Thi, Huu-Huy Nguyen, Minh-Khoi Le, Quoc-Trung Lam, Tuan-Anh Nguyen

**Affiliations:** a University of Medicine and Pharmacy at Ho Chi Minh City, Ho Chi Minh City, 700000, Viet Nam; b University Medical Center Ho Chi Minh City, Ho Chi Minh City, 700000, Viet Nam; c Molecular Biomedical Center, University Medical Center Ho Chi Minh City, Ho Chi Minh City, Viet Nam

**Keywords:** *EGFR*, *KRAS*, Mutation, Non-small cell lung cancer, Vietnamese patients, Tyrosine kinase inhibitors

## Abstract

**Introduction:**

This study examines the genetic and clinical profiles of Vietnamese patients with non-small cell lung cancer (NSCLC), focusing on mutations in seven driver genes: *EGFR*, *KRAS*, *NRAS*, *BRAF*, *ALK*, *ROS1*, and *PIK3CA*. The goal is to identify mutation patterns and their correlations with clinical factors, thereby informing personalized treatment strategies.

**Materials and methods:**

A cross-sectional study of 299 NSCLC patients at the University Medical Center, Ho Chi Minh City (2019–2022) recorded demographics, smoking history, and tumor stage. Pre-treatment samples were analyzed via massively parallel sequencing, and survival analysis assessed the impact of *EGFR/KRAS* mutations on survival and TKI response.

**Results:**

Most patients (88.6 %) were diagnosed at stage IV. *EGFR* mutations were found in 43.5 % of cases, predominantly in female non-smokers, while *KRAS* mutations (15.4 %) were more common in male smokers. *EGFR* exon 19 deletions (46.3 %) and L858R (39.0 %) were the most frequent, with *KRAS* G12C (29.8 %) as the dominant variant. *EGFR*-mutant patients treated with TKIs had significantly longer survival (p = 0.027); however, no survival difference was observed between the *EGFR*- and *KRAS*-mutated groups. Co-mutations (3.7 %) were rare but may indicate resistance. Logistic regression confirmed *EGFR* mutations' association with female non-smokers and *KRAS* mutations with male smokers.

**Conclusions:**

Genetic profiling in Vietnamese NSCLC patients reveals a high prevalence of actionable driver mutations, supporting the integration of routine molecular testing into NSCLC management. *EGFR*-mutated patients derive significant benefits from TKI therapy, underscoring the importance of personalized treatment strategies. Further research is needed to investigate resistance mechanisms and refine targeted therapeutic approaches.

## Highlights

• Most patients exhibit mutations, with 66.9 % having a mutation in a single gene and 3.7 % showing co-mutations.
• Adenocarcinoma: most common histological type (93.3 %); Squamous Cell Carcinoma: 5.0 %.
• *EGFR* mutations were present in 43.5 % of patients, mainly in female non-smokers, while *KRAS* mutations were found in 15.4 % of cases, primarily in male smokers.
• No co-occurrence of *EGFR* and *KRAS* mutations was observed.
• EGFR-mutated patients who received TKIs exhibited significantly prolonged survival (p = 0.027).

## 1 Introduction

Lung cancer remains the leading cause of mortality, accounting for an estimated 1.8 million deaths, which constituted 18 % of all cancer-related deaths [1]. In Vietnam, approximately 26,000 cases of lung cancer are reported annually, and sadly, around 24,000 of these cases result in fatalities [2]. Consequently, lung cancer stands as the second deadliest disease in the nation, demanding urgent medical attention. The two most common subtypes of lung cancer are small-cell lung carcinoma (SCLC), comprising 20 % of cases, and non-small-cell lung carcinoma (NSCLC), accounting for 80 % of cases [3]. NSCLC has been further categorized into three distinct subtypes: squamous-cell carcinoma, adenocarcinoma, and large-cell carcinoma [4].

Overall, patients with late-stage NSCLC are typically treated with systemic or palliative therapies, such as chemotherapy, radiation therapy, or a combination of both. The 5-year overall survival rates for NSCLC vary based on the stage of the cancer: 26 % for stage IIIb, 10 % for stage IVa, and 1 % for stage IVb [5]. Roughly 80 % of lung cancer patients are diagnosed at an advanced stage, where surgery offers little significant benefit [6]. Previously, patients with advanced stages of NSCLC were often considered less responsive to effective treatments due to poor prognoses. However, advancements in science and technology have enabled the identification of specific gene mutations and the use of targeted medications for treatment. Targeted therapy, which focuses on specific molecular pathways, has emerged as a promising approach. However, its effectiveness hinges on accurately identifying lung cancer cells, with oncogenic driver genes playing a pivotal role [7]. According to the National Comprehensive Cancer Network Guidelines for NSCLC (version 4.2020), it is recommended that all patients with advanced non-squamous NSCLC undergo testing for crucial driver genes, including *EGFR*, *ALK*, *BRAF*, and *ROS1*, regardless of clinical variables [8].

Most data available in publicly accessible databases, such as The Cancer Genome Atlas, come from prospective studies conducted in Caucasian cohorts. As a result, there is a high likelihood of underestimating the impact of NSCLC variations across different nations. GLOBOCAN statistics indicate that despite similar mortality rates, the incidence of lung cancer is significantly higher in Vietnam (14.5 %) compared to the global average (11 %) [9].

In our recent research, conducted at the prestigious University Medical Center in Southern Vietnam, we successfully utilized the "next generation sequencing" technique to identify genetic alterations in seven essential driver genes - *EGFR*, *KRAS*, *NRAS*, *BRAF*, *ALK*, *ROS1*, and *PIK3CA* - in tumor tissues from 299 cases of NSCLC. To our knowledge, this is one of the largest cohorts of NSCLC patients in Vietnam analyzed for seven major driver gene mutations. Moreover, Southeast Asian populations are underrepresented in global mutation profiling studies; our study helps fill this gap. Our analysis also revealed strong correlations between the occurrence of these mutations and various clinical characteristics within the Vietnamese cohort. Furthermore, we thoroughly evaluated current treatment methods for NSCLC. Additionally, we have been monitoring the survival rates of lung cancer patients who have received specialized therapy for one year, and our statistics have revealed some significant findings.

## 2 Materials and methods

### 2.1 Study design

Throughout every procedure, we meticulously adhered to applicable instructions and proper regulations. A total of 299 formalin-fixed paraffin-embedded tumor samples from NSCLC patients under the care of the University Medical Center were comprehensively examined. Hematoxylin and eosin staining were employed to identify tissue regions containing at least 20 % tumor cells. Before participation, each research participant provided their full consent. Testing for gene mutations was carried out only with explicit permission from the patient or their family, and all data collected was encrypted to safeguard patient privacy.

### 2.2 Treatment history of patients

Establishing a baseline genomic profile before treatment is crucial to accurately assess mutation patterns without interference from therapy-induced resistance. Genetic profiling before treatment was administered to the patients, ranging from first-line chemotherapy and radiation to advanced targeted therapies, such as osimertinib (n = 42), ceritinib (n = 5), pembrolizumab (n = 1), and bevacizumab-afatinib (n = 2). At the time of genomic analysis, patients had not received any therapy. Treatment regimens were later documented based on patient medical records from the University Medical Center in Ho Chi Minh City.

### 2.3 DNA isolation

We employed two different techniques to extract DNA from formalin-fixed paraffin-embedded specimens and quantify them: the QIAamp DNA FFPE Tissue Kit (Qiagen, USA) and the QuantiFluor dsDNA system (Promega, USA).

### 2.4 Massively parallel sequencing

We utilized NEBNext® UltraTM II kits from New England Biolabs, USA, to generate high-quality libraries from various input quantities and fragmentize DNA. We employed the QuantiFluor dsDNA system from Promega, USA, to measure DNA library concentrations. The seven driver genes *EGFR* (ID: 1956), *KRAS* (ID: 3845), *NRAS* (ID: 4893), *BRAF* (ID: 673), *ALK* (ID: 238), *ROS1* (ID: 238), and *PIK3CA* (ID: 5290) were hybridized with xGen Lockdown probes obtained from IDT DNA, USA, with an equal amount of 150 ng for each sample library. Customized probes for intron regions of *ALK* and *ROS1* were created and combined with exon region probes at an equal concentration. Sequencing was performed using the NextSeq system from Illumina, explicitly utilizing the 500/550 High Output kits Version 2 (150 cycles). Illumina's 550 system was used, aiming for a minimum target coverage of 100x. In cases where the mean coverage in the targeted areas fell below 100x, additional sequencing was conducted to raise the mean coverage to the anticipated range. Across all samples, the average coverage in the target regions was approximately 129x.

### 2.5 Variant calling using Mutect2 and Factera

**Fig. 1:**
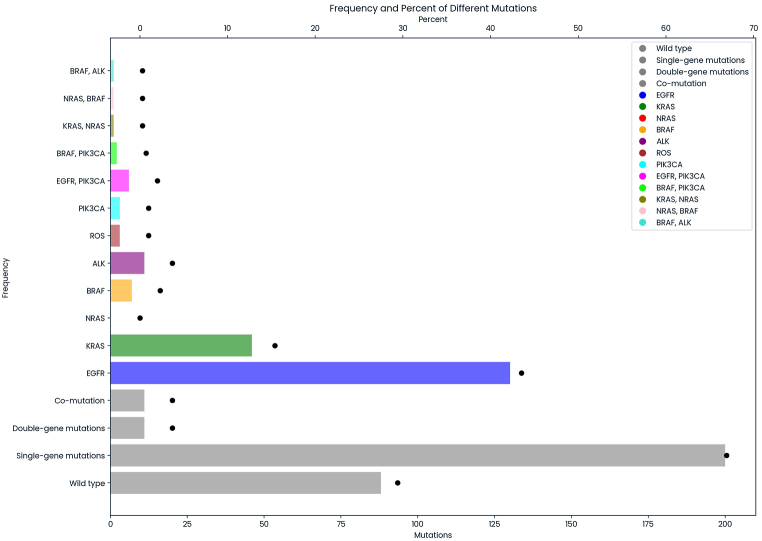
The genetic variation within seven crucial driver genes among 299 Vietnamese NSCLC patients. The bars represent frequencies. The dots represent the percent.

Dual indicators in the P7 and P5 primers were used to barcode each FFPE specimen. The Bcl2fastq package from Illumina was employed to generate paired-end (PE) reads, which were subsequently aligned to the hg38 reference genome using the BWA package [10]. Duplicate reads were identified and labeled using the MarkDuplicates tool from the Picard program (http://broadinstitute.github.io/picard/) or the Mutect2 package [11]. Furthermore, a custom pipeline incorporating the BWA, Picard, and Samtools programs was developed to facilitate these analytical stages [12]. Factera v1.4.4 was utilized with standard settings to detect fusion variants to identify *ALK* and *ROS1* rearrangements [13]. Our targeted capture sequencing method accurately identified mutations in FFPE tissue samples with a variant allele frequency (VAF) greater than 4 % [14]. Each gene investigates different types of mutations, including point mutations, deletions, and short insertions (under 20 nucleotides) in the coding region and adjacent areas near the intron (−20/+10 nucleotides from the exon) as follows: L858 (R, Q)/L861 (P, Q, R)/G719 (A, D, S, C, R, X)/S768I/L747S/A750P/Exon 19 deletion/Exon 20 insertion/T790M for *EGFR*, G12 (A, D, V) or G12 (S, R, C)/G13 (A, D, V) or G13 (S, R, C)/Q61 (K, E, P, L, R, H) or *KRAS*, G12 (A, D, V) or G12 (S, R, C)/G13 (A, D, V) or G13 (S, R, C)/Q61 (K, E, P, L, R, H) for *NRAS*, V600E for *BRAF*, *ALK*-*EML4* fusion, *ALK*-*HIP1*, *PHACTR1* fusion, G1202R for *ALK*, *CD74*, *SLC34A2*, *SDC4*, *EZR*, *TPM3*, Fig. 1, *LRIG3*, *CCDC6*, *KDELR2*, *LRIG3*, *TPD52L1* fusions for *ROS1*, H1047 (R, L) and E545K for *PIK3CA*.

### 2.6 Statistical analysis

Descriptive statistics were used for categorical variables (frequencies and percentages). Statistical analysis using either the Chi-squared test (for sample sizes >5) or Fisher's exact test (for sample sizes ≤5) was employed to determine the *p*-value. The Monte Carlo simulation for larger tables with minor frequencies. Univariate and multivariate analyses assessed the association between clinical characteristics and the frequency of mutations in NSCLC driver genes, especially *EGFR* and *KRAS* mutations. The survival analysis includes three main aspects. First, the Kaplan-Meier analysis assessed survival outcomes of *EGFR* and *KRAS* mutation status and tyrosine kinase inhibitor treatment. Second, the log-rank test was conducted to determine whether survival distributions differed between groups. Finally, the Cox proportional hazards model was used to identify factors influencing survival time. A p-value of <0.05 was considered statistically significant. Statistical analysis was performed using SPSS 26 software.

## 3 Results

### 3.1 Patient characteristics

Our study involves a larger cohort with 299 patients compared to previous local studies, such as those by Dang et al. [14]. This provides a more robust data set for analyzing mutation patterns and their implications for treatment outcomes. Moreover, our research utilizes massively parallel sequencing to identify mutations, offering detailed and extensive genetic profiling compared to Vu et al. [15], which employed Sanger sequencing, a less comprehensive method in mutation detection.

The study cohort includes slightly more males (54.8 %) than females (45.2 %). The population is relatively evenly distributed across age groups, with fewer individuals over 61 (55.9 %) than those aged 61 or younger (44.1 %). A significant portion of the population has unknown smoking (21.4 %) and drinking habits (46.1 %). Adenocarcinoma (AC) is the most common histological type (93.3 %), followed by Squamous Cell Carcinoma (SCC) at 5.0 %. The majority of patients are diagnosed at stage IV (88.6 %). A total of 52.5 % of patients are treatment-naïve (Table 1).

**Table 1:** Key clinical and pathological characteristics observed in 299 Vietnamese patients with NSCLC.

Clinical characteristics		N	%
Gender	Female	135	45.2
	Male	164	54.8
Age	≤61	132	44.1
	>61	167	55.9
Smoking status	Yes	81	27.1
	No	154	51.5
	Unknown	64	21.4
Drinking habits	Yes	37	12.4
	No	124	41.5
	Unknown	138	46.1
Histology	AC	279	93.3
	SCC	15	5.0
	Others or unknown	5	1.7
Tumor Stage	II	14	4.7
	III	20	6.7
	IV	265	88.6
Treatment information	Chemotherapy	41	13.7
	Naïve to treatment	157	52.5
	Palliative care	37	12.4
	Radiation	4	1.3
	Resection	10	3.3
	TKI	50	16.7

N: number of cases; AC: adenocarcinoma; SCC: squamous cell carcinoma.

### 3.2 Mutation frequencies and co-mutations in NSCLC driver genes

The analysis of the occurrence of wild-type and mutated genes within the studied population presents the frequency of mutations across the seven primary driver genes and the specific mutation frequency in cases where co-mutations are observed.

Most patients exhibit mutations, with 66.9 % having mutations in a single gene and 3.7 % showing co-occurring mutations. Only 29.4 % of patients are wild-type. *EGFR* mutation is the most prevalent in 43.5 % of patients, while *KRAS* is detected in 15.4 %. In a minority of cases, concurrent mutations, where patients have mutations in more than one gene, were detected in various gene combinations. Specifically, *EGFR* and *PIK3CA* co-mutations were observed in 2.0 % of cases, while co-mutations involving *BRAF* and *PIK3CA* occurred in 0.7 % of cases. Moreover, *KRAS* and *NRAS* co-mutations were identified in 0.3 % of cases, with NRAS and BRAF co-mutations occurring at the same frequency. Co-mutations involving *BRAF* and *ALK* were also observed in 0.3 % of cases (Fig. 1).

**Fig. 2:**
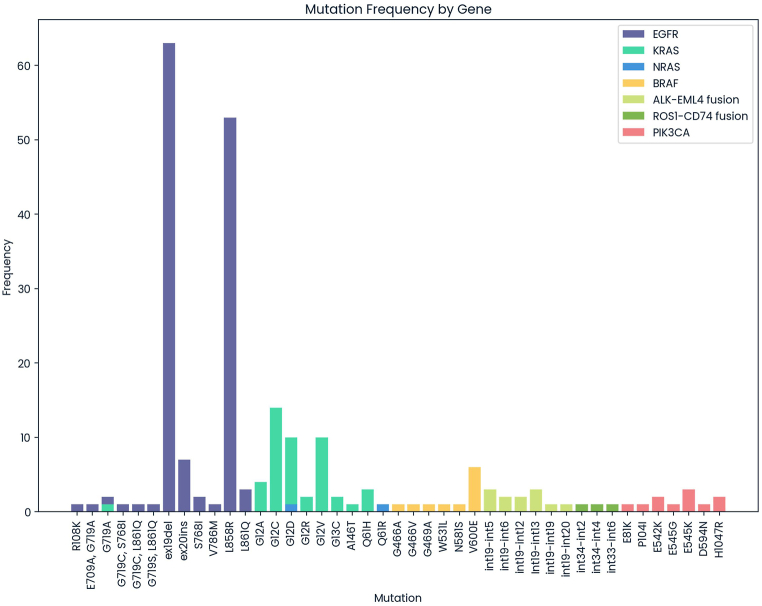
The distribution of mutation subtypes across seven driver genes, namely *EGFR*, *KRAS*, *BRAF*, *NRAS*, *ALK*, *ROS1*, and *PIK3CA*, was analyzed in Vietnamese NSCLC. The mutation frequencies (%) within specific subtypes of these genes were determined as the percentage of mutant cases among the total number of cases with mutations. The bars represent frequencies.

The study population exhibited a variety of *EGFR* mutations, ranging from point mutations to insertions and deletions. Exon 19 deletions (46.3 %) and the L858R mutation (39.0 %) were the most prevalent. Less common mutations included exon 20 insertions (5.1 %) and co-mutations. The frequency and distribution of *KRAS* mutations in the study population revealed a diverse array of mutations across various codons, with G12C (29.8 %), G12D (21.3 %), and G12V (21.3 %) being the most frequent. Rarer mutations, such as G12A, G12R, G13C, A146T, G719A, and Q61H, contributed to the molecular diversity of *KRAS*-mutant cancers. Two *NRAS* mutations, G12D and Q61R, accounted for 50 % of observed mutations. The predominant *BRAF* mutation was V600E, accounting for 54.5 % of BRAF mutations, while other mutations, including G466A, G466V, G469A, W531L, and N581S, each represented 9.1 %. The most common *ALK-EML4* fusion variants were int19-int5 and int19-int13, each at 25.0 %, followed by int19-int6 and int19-int12, at 16.7 % each. Int19-int19 and int19-int20 were less frequent, at 8.3 % each. *ROS1-CD74* fusion variants included int34-int2, int34-int4, and int33-int6, each representing 33.3 % of mutations. *PIK3CA* mutations encompassed variants like E81K, P104I, E542K, E545G, E545K, D594N, and H1047R. E545K (27.3 %) and H1047R (18.2 %) were the most common mutations, followed by E542K (18.2 %), and less frequent mutations, such as E81K, P104I, E545G, and D594N, each at 9.1 % (Fig. 2).

### 3.3 Associations between clinical characteristics and NSCLC driver gene mutations

Table 2 presents the distribution of clinical characteristics by mutation status for seven genes (*EGFR*, *KRAS*, *NRAS*, *BRAF*, *ALK*, *ROS1*, and *PIK3CA*), with p-values indicating statistical significance. For *EGFR*, mutations are significantly more common in females (60.7 % vs. 32.9 % in males; *p* < 0.001) and non-smokers (72.1 % vs. 29.6 % in smokers; *p* < 0.001). Moreover, non-drinkers tend to exhibit a higher prevalence of *EGFR* mutations (53.2 %) compared to drinkers (35.1 %), although this difference is marginally significant (*p* = 0.053). In contrast, *KRAS* mutations are significantly more prevalent in males (23.8 % vs. 5.9 % in females; *p* < 0.001) and smokers (44.4 % vs. 7.1 % in non-smokers; *p* < 0.001), with a significant association also observed with alcohol consumption (*p* = 0.001). *ALK* mutations are notably linked with female gender (6.7 % vs. 0.6 % in males; *p* = 0.041) and with younger patients (7.6 % in individuals ≤61 years vs. 1.2 % in those >61 years; *p* = 0.007), while differences by smoking and alcohol status are not significant. In addition, the prevalence of *ROS1* mutations does not differ significantly by gender, age, smoking, or alcohol consumption. Similarly, mutations in *NRAS, BRAF, and PIK3CA* show no significant associations with these clinical parameters. Analyses by histologic subtype and tumor stage also reveal no significant correlations with mutation status across the examined genes, except for treatment groups, where significant differences were observed for *EGFR* and *KRAS* (*p* < 0.001). Collectively, these results underscore that while specific clinical factors such as gender, age, smoking, and alcohol consumption are robustly associated with the prevalence of mutations in *EGFR*, *KRAS*, and *ALK*, they do not appear to influence the mutation status of *NRAS*, *BRAF*, *ROS1*, or *PIK3CA*.

**Table 2:** The correlation between clinical variables and the occurrence rates of mutations in NSCLC driver genes.

Clinical characteristics		*EGFR*			*KRAS*			*NRAS*			*BRAF*			*ALK*			*ROS1*			*PIK3CA*		
		Yes	No	p	Yes	No	p	Yes	No	p	Yes	No	p	Yes	No	p	Yes	No	p	Yes	No	p
Gender	Female	82	53	<0.001	8	127	<0.001	0	135	0.503	4	131	0.759	9	126	0.041	2	133	0.591	5	130	0.984
	Male	54	110		39	125		2	163		7	157		3	161		1	163		6	158	
Age	≤61	63	69	0.489	22	110	0.689	1	131	1.000	4	128	0.760	10	122	0.007	2	130	0.585	4	128	0.760
	>61	73	94		25	142		1	166		7	160		2	165		1	166		7	160	
Smoking status	Yes	24	57	<0.001	36	45	<0.001	2	79	0.118	1	80	0.662	1	80	0.169	0	81	1.000	3	78	1.000
	No	111	43		11	143		0	154		4	150		8	146		1	153		7	147	
	Unknown	1	63		0	64		0	64		6	58		3	61		2	62		1	63	
Alcohol drinking	Yes	13	24	0.053	14	23	0.001	2	35	0.052	1	36	1.000	0	37	0.120	0	37	1.000	2	35	1.000
	No	66	58		17	107		0	124		4	120		9	115		1	123		7	117	
	Unknown	57	81		16	122		0	138		6	132		3	135		2	136		2	136	
Histology	ACC	130	149	0.315	44	235	0.482	1	278	0.100	9	270	0.413	12	267	1.000	3	276	1.000	10	269	0.444
	SCC	5	10		1	14		1	14		1	14		0	15		0	15		1	14	
	Unknown	1	4		2	3		0	5		1	4		0	5		0	5		0	5	
Tumor stage	II	10	4	0.056	2	12	0.925	0	14	1.000	0	14	0.501	0	14	0.505	0	14	1.000	0	14	0.238
	III	6	14		4	16		0	20		0	20		0	20		0	20		2	18	
	IV	120	145		41	224		2	263		11	254		12	253		3	262		9	256	
Treatment	1	67	90	<0.001	21	136	<0.001	1	156	0.102	8	149	0.288	6	151	0.454	1	156	0.352	2	155	0.163
	2	7	34		17	24		0	41		3	38		0	41		1	40		3	38	
	3	14	23		5	32		0	37		0	37		3	34		1	36		1	36	
	4	1	3		2	2		0	4		0	4		0	4		0	4		0	4	
	5	4	6		2	8		1	9		0	10		0	10		0	10		1	9	
	6	43	7		0	50		0	50		0	50		3	47		0	50		4	46	

The abbreviations ACC and SCC represent adenocarcinoma and squamous carcinoma, respectively. Tumor stage: II, III, IV. Treatment: 1, 2, 3, 4, 5, and 6 are naïve to treatment, chemotherapy, palliative care, radiation, resection, and TKI, respectively.

To assess the potential influence of prior treatments on genomic alterations, we performed subgroup analyses stratifying patients by their treatment histories. This analysis aimed to identify any significant patterns of mutations associated with specific therapies, with a primary focus on secondary resistance mutations in the *EGFR* and *KRAS* genes (Table 3) (see Table 4).

**Table 3:** The frequency of specific mutations stratified by treatment type.

Treatment information	Chemotherapy	Naïve to treatment	Palliative care	Radiation	Resection	TKI
None	12	54	14	1	3	4
*EGFR* ex19del	1	27	8	1	2	21
*EGFR* L858R	3	27	3	–	2	16
*KRAS* G12C	5	8	1	–	–	–
*KRAS* G12D	3	5	1	1	–	–
*EGFR* Exon20ins	1	4	–	–	–	1
*BRAF* V600E	–	4	–	–	–	–
*KRAS* G12A	–	4	–	–	–	–
*KRAS* g12v	5	2	3	–	–	–
*KRAS* Q61H	1	2	–	–	–	–
*ALK*-*EML4* (int19-int6)	–	2	–	–	–	–
*ALK*-*EML4* fusion (int19-int13)	–	2	–	–	–	–
*EGFR* S768I	–	2	–	–	–	–
*EGFR* G719A	–	1	–	–	–	1
*PIK3CA* D594N	–	1	–	–	–	–
*ROS1*-*CD74* fusion (int34-int4)	–	1	–	–	–	–
*ALK*-*EML4* fusion (int19-int20)	–	1	–	–	–	–
*BRAF* G466V	–	1	–	–	–	–
*BRAF* N581S	–	1	–	–	–	–
*BRAF* W531L, *ALK*-*EML4* fusion (int19-int13)	–	1	–	–	–	–
*NRAS* G12D, *BRAF* G469A	–	1	–	–	–	–
*EGFR* Exon20ins, *PIK3CA* E545G	–	1	–	–	–	–
*EGFR* G719A, E709A	–	1	–	–	–	–
*EGFR* G719C, S768I	–	1	–	–	–	–
*EGFR* G719S; L861Q	–	1	–	–	–	–
*EGFR* L861Q, G719C	–	1	–	–	–	–
*EGFR* R108K	–	1	–	–	–	–
*ALK*-*EML4* fusion (int19-int5)	–	–	2	–	–	1
*ALK*-*EML4* fusion (int19-int12)	–	–	1	–	–	1
*ALK*-*EML4* fusion (int19-int19)	–	–	–	–	–	1
*EGFR* ex19del, *PIK3CA* E545K	–	–	–	–	–	1
*EGFR* ex19del, *PIK3CA* H1047R	–	–	–	–	–	1
*EGFR* L858R, *PIK3CA* H1047R	–	–	–	–	–	1
*EGFR* L858R, *PIK3CA* p104l	–	–	–	–	–	1
*KRAS* g12r	–	–	–	1	1	–
*PIK3CA* E542K	–	–	–	–	1	–
*KRAS* G13C, *NRAS* Q61R	–	–	–	–	1	–
*EGFR* L861Q	1	–	2	–	–	–
*ROS1*-*CD74* (int33-int6)	–	–	1	–	–	–
*EGFR* ex19del, *PIK3CA* E81K	–	–	1	–	–	–
*PIK3CA* E545K	1	–	–	–	–	–
*ROS1*-*CD74* (int34-int2)	1	–	–	–	–	–
*BRAF* G466A	1	–	–	–	–	–
*BRAF* V600E, *PIK3CA* E542K	1	–	–	–	–	–
*BRAF* V600E, *PIK3CA* E545K	1	–	–	–	–	–
*KRAS* A146t	1	–	–	–	–	–
*KRAS* G13C	1	–	–	–	–	–
*KRAS* G719A	1	–	–	–	–	–
*EGFR* V786M	1	–	–	–	–	–

None: No mutation in the penal was detected. Int: insertion. (−) no mutation exists. The number represents the frequency of specific mutations.

**Table 4:** Logistic regression analysis of clinical factors associated with *EGFR* and *KRAS* mutation status.

Clinical characteristics	*EGFR*			*KRAS*		
	*p*	OR	95 % CI	*p*	OR	95 % CI
Gender	0.607	0.765	0.276–2.125	0.075	3.797	0.875–16.47
Age	0.835	0.917	0.406–2.071	0.273	1.900	0.603–5.987
Smoking status	0.128	0.399	0.122–1.304	0.084	4.020	0.830–19.47
Alcohol drinking	0.895	1.087	0.314–3.768	0.892	0.892	0.190–4.180
Histology	0.299	0.423	0.083–2.146	–	–	–
Tumor stage III	0.021	11.07	1.443–84.86	0.101	0.106	0.007–1.543
Tumor stage IV	0.601	1.536	0.308–7.655	0.191	0.281	0.042–1.881
Chemotherapy	0.004	0.163	0.048–0.559	0.004	8.536	2.003–36.38
Palliative care	0.213	0.498	0.166–1.492	0.496	1.691	0.372–7.681
Radiation	0.557	0.448	0.031–6.538	0.029	43.77	1.466–1307
Resection	0.632	0.651	0.113–3.760	0.724	0.618	0.043–8.949
Tyrosine Kinase Inhibitor	0.001	7.300	3.378–22.40	–	–	–

OR: Odds ratio; Reference categories: Tumor stage II, Naïve to treatment.

Patients with different genetic mutations received varying treatments, which included chemotherapy, naive to treatment, palliative care, radiation, resection, and targeted kinase inhibitors (TKIs). Patients whose testing panel detects no mutation may undergo various interventions, likely based on other clinical factors outside the detected gene mutations.

*EGFR* mutations, such as ex19del and L858R, are associated with a higher proportion of patients receiving TKI treatments (21 % and 16 %, respectively), which aligns with the finding that *EGFR* mutations often respond well to TKIs. These mutations are also notable in patients receiving chemotherapy and those considered naive to treatment, indicating the prevalence and importance of these mutations in treatment decisions. *KRAS* mutations, such as G12C and G12D, exhibit lower rates of receiving TKIs, consistent with KRAS mutations typically associated with poorer outcomes and reduced responsiveness to TKIs. Many patients with various mutations are treatment-naive, which could reflect newly diagnosed cases or those waiting to begin a treatment regimen. Some mutations with complex or multiple genetic alterations, such as EGFR Exon20ins and PIK3CA E545G, are found in very few patients, often only one or two, suggesting that these are rare or less commonly targeted for specific treatments. Palliative care numbers are generally low, which might indicate either a lower rate of end-stage patients in this dataset or a focus on more aggressive treatment strategies in this particular clinical setting.

### 3.4 Logistic regression analysis of clinical factors influencing EGFR and KRAS mutations

In this study on *EGFR* mutation status, significant findings include that Stage III patients are significantly more likely to exhibit positive *EGFR* mutations with an odds ratio (OR) of 11.07 (p = 0.021), suggesting advanced cancers are more mutation-prone. There is a notable negative correlation between chemotherapy and EGFR mutations (OR = 0.163, p = 0.004), indicating that chemotherapy is more often prescribed to patients without these mutations. This is likely because patients with mutations are prioritized for other treatments, such as tyrosine kinase inhibitors (TKIs). TKIs exhibit a strong positive association with EGFR mutations (OR = 7.3, p = 0.001), confirming that patients with these mutations are significantly more likely to receive TKI treatment. Other clinical factors like gender, age, smoking, and alcohol consumption did not demonstrate a significant relationship with *EGFR* mutation status (p > 0.05), highlighting that the most important predictors of mutation status are cancer stage and treatment type.

In the analysis of *KRAS* mutation status, the results demonstrate statistically significant associations for patients undergoing chemotherapy (p = 0.004, OR = 8.536), indicating that they are approximately 8.5 times more likely to have a positive *KRAS* mutation than the naive-to-treatment group. This pattern suggests that *KRAS* mutation carriers, who typically respond poorly to TKI intended for *EGFR*/*ALK*-positive cancers, often receive chemotherapy or radiation. Radiation treatment also shows a strong positive correlation with *KRAS* mutations (p = 0.029, OR = 43.77), though the wide confidence interval suggests a small or unevenly distributed sample. This indicates that patients with *KRAS* mutations are significantly more likely to require radiation, either as a supplementary treatment or for symptom relief. Other variables such as gender, age, smoking, and tumor stage did not show significant results (p > 0.05), although trends indicate that *KRAS* mutations are more prevalent among males and smokers. The negligible response of *KRAS* mutation-positive patients to TKIs is also highlighted, suggesting a minimal use of TKIs in this group. Overall, the significant links between chemotherapy, radiation, and KRAS mutations align with the biological behavior of *KRAS*-mutant lung cancer, underlining the necessity for alternative treatments like chemotherapy and radiation in these patients.

### 3.5 Survival analysis by EGFR and KRAS mutation status and TKI treatment

**Fig. 3:**
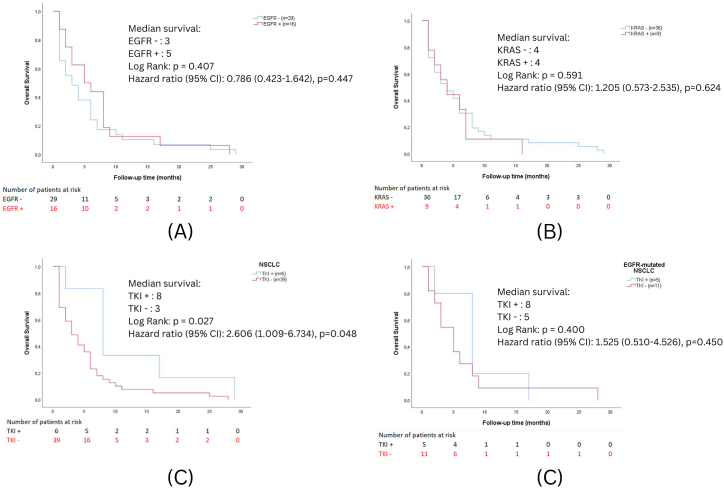
Overall survival varies according to *EGFR* (5A) and *KRAS* (5B) mutation status and *EGFR* tyrosine kinase inhibitors (TKI) treatment in NSCLC (5C) and *EGFR*-mutated NSCLC (5D) patients.

In the analysis stratified by *EGFR* status (n = 45), the median survival was 3 months for the *EGFR*‐negative group and 5 months for the *EGFR*‐positive group. Still, this difference was not statistically significant (p = 0.407) by either the log-rank test or Cox regression (HR = 0.786, p = 0.447). A similar pattern was observed when stratifying by *KRAS* status: 45 patients were included, and both the *KRAS*‐negative and *KRAS*‐positive groups had a median survival of four months, with no significant difference in survival (p = 0.591) and an HR of 1.205 (p = 0.624). In contrast, when comparing TKI recipients (n = 6) with non‐TKI recipients (n = 39), the Kaplan-Meier analysis showed a significantly longer median survival for the TKI group (8 vs. 3 months, p = 0.027). However, the Cox model yielded a hazard ratio of 2.606 (p = 0.048), which may be attributed to confounding factors such as disease severity or the small sample size. Focusing specifically on the 16 EGFR‐mutated NSCLC patients, the Cox regression indicated a hazard ratio of 1.525 (p = 0.450) for TKI use, a finding that was not statistically significant. The wide confidence interval (0.510–4.562) and small sample underscore the limited power of this subgroup analysis. Overall, the unadjusted Kaplan–Meier results for TKI use in the entire 45-patient cohort suggest a survival benefit. In contrast, the hazard ratio and smaller subgroup analysis raise questions that require cautious interpretation (Fig. 3).

## 4 Discussion

Establishing a solid genetic predisposition to lung cancer could be clinically valuable for stratifying populations for effective treatment and primary prevention. Our study provides detailed insights into the prevalence and types of mutations across various demographic groups, including age, smoking status, and gender, offering valuable data for personalized medicine in the treatment of NSCLC. We identified significant correlations between the presence of *EGFR*, *KRAS*, and *ALK* mutations and clinical characteristics such as gender, age, smoking habits, and alcohol consumption in the Vietnamese cohort. Additionally, we assessed the effectiveness of current NSCLC treatments and monitored the survival rates of lung cancer patients after one year of specialized therapy. To the best of our knowledge, this represents one of the most extensive Vietnamese NSCLC cohorts examined for seven primary driver mutations. Furthermore, given the limited representation of Southeast Asian populations in global genomic studies, our research provides important insights to help bridge this disparity.

Among 299 Vietnamese NSCLC patients, the majority (88.6 %) presented with advanced disease at the time of diagnosis. Adenocarcinoma was the most common histological type, accounting for 93.3 % of cases. Additionally, more than 50 % of patients reported not receiving specific therapy for their condition. However, some patients with stage II NSCLC were enrolled and received targeted therapy if they had gene mutations. This is a common scenario in our hospital's clinical setting, as physicians often recommend TKIs as adjuvant therapy following complete surgical resection for stage II patients, mainly when specific genetic mutations are present. This approach is generally considered when patients have the financial resources to afford TKI therapy, given that healthcare reimbursement for these treatments is often limited.

Furthermore, as mentioned in our study, the decision to forgo targeted therapy for some patients often arises from financial constraints or deteriorating health. In such cases, palliative care may be deemed a more suitable option. These factors significantly impact clinical decision-making, highlighting the need for a personalized approach. These data provide valuable insights into the demographics, disease characteristics, and treatment landscape of the study population.

### 4.1 Actionable genomic insights in Vietnamese NSCLC

We employed targeted next-generation sequencing to analyze seven actionable driver genes - *EGFR*, *KRAS*, *NRAS*, *BRAF*, *ALK*, *ROS1*, and *PIK3CA* - in formalin-fixed, paraffin-embedded tumor specimens from Vietnamese NSCLC patients. This approach is more comprehensive than earlier studies, which focused primarily on *EGFR* [15,16]. Our findings confirmed *EGFR* as the most prevalent mutation, aligning with previous research [14,[17], [18], [19], [20]], while *KRAS* emerged as the second most frequent [14,19,21]. These results underscore the global impact of racial, genetic, and environmental factors on mutation variability [17,18].

Recent investigations into the mutation spectrum of NSCLC have highlighted notable ethnic and regional variations, particularly in the incidence of *EGFR* and *KRAS* mutations. In our cohort, the *EGFR* mutation rate reached 43.5 %, aligning with findings in other East Asian populations - such as China (30–50 %) [22] and Japan (around 43.6 %) [23] - but substantially exceeding the figures reported in Caucasian (9.8–17.5 %) [24,25] and African-American groups (4.8–19 %) [26,27] (Table 5). These higher *EGFR* frequencies among Vietnamese and other East Asians suggest a strong influence from genetic predisposition, lifestyle, and environmental factors unique to these regions. Smoking habits may play an additional role, as reduced smoking prevalence or patterns could partially explain the relatively high occurrence of *EGFR* mutations in these populations.

**Table 5:** EGFR and KRAS mutation frequencies in NSCLC by ethnicity and region.

Study population	EGFR mutation (%)	KRAS mutation (%)	Source
Vietnamese	43.5	15.4	This study
Other Vietnamese	35.0–35.4	22.6–23.0	[2,14]
Chinese	30.0–50.0	8.3	[22,52]
Japanese	43.6	9.3	[23]
Korean	17.4–51.3	5.2–5.8	[[53], [54], [55]]
Caucasian	9.8–17.5	23.0–35.0	[24,25,28]
African-American	4.8–19.0	17.0–25.0	[26,27]

In contrast, *KRAS* mutations were observed in 15.4 % of our study cohort, which is significantly lower than the 23–35 % (Table 5) found in Western cohorts [24,28]. This trend reflects an inverse relationship between *EGFR* and *KRAS* mutations. While patients in East Asian countries generally exhibit elevated *EGFR* mutation rates, they tend to have fewer *KRAS* mutations compared to Western populations. Nonetheless, some variation exists even within Vietnam itself. Compared to two prior studies in the same country [2,14]*, EGFR* mutation rates in this cohort were higher, whereas *KRAS* frequencies were lower (Table 5). Such discrepancies suggest that factors such as local environment, healthcare infrastructure, and genetic diversity within an ethnic group may substantially influence the prevalence and detection of specific mutations.

These findings underscore the importance of establishing ethnic-specific genomic databases and treatment guidelines, which are vital for ensuring equitable access to precision medicine. Routine molecular profiling of NSCLC tumors helps clinicians identify actionable mutations, thereby informing targeted therapies. Asian patients, who often have higher *EGFR* mutation rates, frequently benefit from *EGFR* tyrosine kinase inhibitors, a mainstay treatment in regions such as Vietnam, China, Japan, and Korea. Conversely, Western patients who show an elevated incidence of *KRAS* mutations may require alternative therapeutic approaches. Novel agents targeting *KRAS* G12C, for example, offer promising avenues for patients harboring *KRAS* mutations; however, they remain under investigation and are not as commonly indicated for East Asian populations.

Recognizing these molecular distinctions is crucial for optimizing care in NSCLC, highlighting the need for personalized screening, prevention, and therapeutic strategies. By accounting for genetic, environmental, and lifestyle factors, clinicians can better identify which treatments are most likely to be effective for specific patient populations. In an era where personalized medicine is increasingly driving oncology research, documenting ethnic and regional differences offers a roadmap to expand the benefits of targeted therapies worldwide.

Further analysis revealed that *EGFR* mutations were notably higher in female non-smokers, consistent with established correlations between *EGFR*, gender, and smoking status [14,18,19,29]. Meanwhile, *KRAS* mutations were more common among smokers, reflecting the strong link between cigarette smoking and *KRAS* alterations [14,24,30,31]. These observations advance our understanding of the molecular landscape of Vietnamese NSCLC, emphasizing the importance of actionable genomic profiling in guiding personalized therapies and informing future targeted research.

### 4.2 Comprehensive analysis of key oncogenic mutations in NSCLC

Identifying diverse oncogenic drivers underscores the complexity of NSCLC and highlights the need for comprehensive genomic profiling to guide personalized treatment [32]. Common *EGFR* mutations (e.g., ex19del and L858R) correlate strongly with favorable responses to inhibitors like gefitinib and erlotinib [15]. By contrast, *KRAS* mutations (particularly G12C, G12D, and G12V) often predict poor prognosis and resistance to EGFR inhibitors, emphasizing the importance of alternative therapeutic strategies [33]. Notably, the emergence of KRAS G12C inhibitors - such as sotorasib and adagrasib - offers significant therapeutic potential; these agents are now approved for second-line use in advanced or metastatic NSCLC and highlight the importance of comprehensive molecular testing to guide therapy selection [34].

Although less frequent, *NRAS* mutations (∼1 %) share signaling pathways with *KRAS*, potentially conferring sensitivity to MEK inhibitors like selumetinib and trametinib [35]. *BRAF* mutations, including the well-characterized V600E variant, can be targeted with combined BRAF/MEK inhibition (dabrafenib plus trametinib), thereby improving treatment efficacy [36]. ALK-EML4 and ROS1-CD74 fusions also play critical roles in tumorigenesis, with established targeted options (e.g., crizotinib and newer ALK inhibitors) that significantly enhance patient outcomes [[37], [38], [39]]. Finally, *PIK3CA* mutations, such as E542K, E545K, and H1047R, underscore the relevance of the PI3K/AKT/mTOR pathway in NSCLC and hold promise for future targeted therapies [40,41]. Overall, these findings underscore the importance of routine biomarker testing, preferably via next-generation sequencing, and the integration of emerging treatments to address the molecular heterogeneity of NSCLC.

### 4.3 Distribution of driver mutations across lung cancer histological types

Lung cancers are primarily classified as non-small cell lung cancer (NSCLC, ∼80 %) and small cell lung cancer (SCLC, ∼20 %) [3]. Our histological assessment identified *EGFR* and *KRAS* as predominant driver genes, consistent with a report showing at least eight pathological subtypes in lung adenocarcinoma [18]. Variations in smoking habits and racial backgrounds may explain the genetic disparities observed across histological groups [37], and most research indicates *EGFR* and *KRAS* mutations rarely coexist [38].

In this study, adenocarcinoma showed a higher frequency of mutations than squamous cell carcinoma or cancers of unknown histology [39]. The predominance of *EGFR* mutations in adenocarcinoma highlights its potential as a therapeutic target for EGFR inhibitors [40]. The absence of *ALK* and *ROS1* rearrangements in squamous cell carcinoma suggests a lesser role for these fusions in that subtype. However, a rare case of lung squamous cell carcinoma with a *ROS1* rearrangement showed marked sensitivity to crizotinib [41]. These findings underscore the need for comprehensive genomic profiling to guide personalized therapy.

Few mutations were observed in the unknown histology group, indicating a need for further classification to determine their clinical implications. Less common mutations, such as *NRAS* and *PIK3CA*, also warrant additional investigation in adenocarcinoma and squamous cell carcinoma, potentially through targeted clinical trials [42]. Ultimately, understanding the mutation landscape across different histological types can foster more effective, patient-specific treatment strategies [43].

### 4.4 Co-mutation profiles and the impact of targeted treatment

Co-mutation profiling is critical for designing targeted therapies that may address multiple mutations simultaneously. Our study observed a lower co-mutation rate (3.7 %) than another study reporting 12.3 % in stage IV disease [39]. Notably, co-mutations in *EGFR* and *PIK3CA* could reflect crosstalk between the EGFR and PI3K-AKT-mTOR pathways, potentially indicating poor prognosis in advanced *EGFR*-mutant lung adenocarcinomas [40,41]. Co-mutations in *BRAF* and *PIK3CA* may signify an aggressive phenotype, as both mutations correlate with poor outcomes in colorectal cancer [42,43]. Similarly, *KRAS* and *NRAS* co-mutations may activate overlapping downstream signaling pathways (e.g., RAF-MEK-ERK and MEK-ERK, respectively) [44,45], though their exact clinical impact remains unclear.

*BRAF* mutations are relatively rare in NSCLC (2.3 % of lung adenocarcinoma) and often appear in never-smokers [46]. Thus, *NRAS*-*BRAF* co-mutations are even rarer and may indicate complex tumor heterogeneity [47]. Some studies have reported co-mutations in *BRAF* and *ALK* [48], suggesting possible cross-regulation between the MAPK and ALK pathways. Although *KRAS*-*EGFR* co-mutations have been reported elsewhere, their incidence is less than 1 %, and none were identified in our cohort [49]. Overall, these patterns highlight the disease's molecular diversity and complexity. Further research is needed to clarify the clinical significance of these co-mutations and guide personalized treatment strategies.

Additionally, interpreting genomic data in patients with diverse treatment histories poses challenges, as prior therapies can induce secondary mutations like *EGFR* T790M. This study acknowledges that variability in treatment backgrounds limits the generalizability of findings. Future research with more uniform cohorts or statistical controls for prior treatments could better isolate the effects of specific genomic alterations on treatment response and disease progression. Notably, resistance mutations (e.g., secondary *EGFR* mutations) were more common in patients previously exposed to TKIs, underscoring the selective pressure exerted by targeted therapies [50,51].

### 4.5 Overall survival to *EGFR* and *KRAS* mutation status and tyrosine kinase inhibitor treatment

Unlike many studies [15,16], our approach extends beyond simple mutation detection to examine relationships with progression-free survival and overall outcomes in advanced-stage III and IV NSCLC. By correlating specific mutations with survival and treatment responses, we provide practical insights for personalized therapy - particularly relevant in Vietnam, where such detailed data have been scarce.

Our findings reveal that *EGFR* and *KRAS* mutations are correlated with declining cumulative survival (Fig. 3A and B). Although *KRAS*-mutant patients generally have lower survival rates than non-mutants, this difference is less pronounced than in *EGFR*-mutant cases. Consistent with previous observations, *EGFR* mutations are more common in older patients with worse survival [47]. Moreover, *EGFR* and *KRAS* independently affect outcomes in advanced NSCLC treated with *EGFR*-TKIs, with *EGFR* status influencing progression-free and overall survival. In contrast, KRAS status primarily affects overall survival [48].

Among all NSCLC patients in our study, those receiving TKI therapy showed higher mean and median survival times (Fig. 3C). However, in the *EGFR*-mutant subgroup, the TKI and non-TKI groups had similar survival curves (Fig. 3D), suggesting that *EGFR* mutations do not always predict TKI benefits [38]. This discrepancy could be attributed to geography, ethnicity, treatment practices [49], sample size, or patient demographics.

Despite these nuances, our results illuminate the impact of TKI therapy on Vietnamese patients with advanced NSCLC and lay the groundwork for future clinical trials of targeted therapies. Additional research is needed to clarify the efficacy of TKIs in *EGFR*-mutant patients, as evidenced by a recent study in China, which demonstrated significantly better survival among *EGFR*-TKI-treated patients [50]. Our analysis highlights the importance of genomic profiling and its correlation with survival in informing treatment decisions and enhancing cancer care in Vietnam.

## 5 Conclusion

The study reveals a high prevalence of advanced-stage adenocarcinoma in Vietnamese NSCLC patients. It identifies strong links between *EGFR* and *KRAS* mutations with factors such as gender and smoking status. *EGFR* mutations are predominantly found in female non-smokers, whereas *KRAS* mutations are more common among male smokers. The use of *EGFR* inhibitors as targeted therapies has demonstrated significant survival benefits, emphasizing the need for personalized treatment plans based on detailed genomic profiling. Additionally, the research highlights the intricate relationship between a patient's treatment history and genomic alterations, stressing the importance of careful analysis in determining the impact of previous treatments. This necessitates further studies to understand these interactions better and enhance personalized treatment approaches in NSCLC.

## CRediT authorship contribution statement

**Hoang-Bac Nguyen:** Writing – review & editing, Supervision, Resources, Project administration, Investigation, Funding acquisition, Conceptualization. **Bang-Suong Nguyen-Thi:** Writing – original draft, Supervision, Resources, Project administration, Investigation, Funding acquisition, Conceptualization. **Huu-Huy Nguyen:** Visualization, Validation, Formal analysis. **Minh-Khoi Le:** Software, Methodology, Data curation. **Quoc-Trung Lam:** Software, Methodology, Formal analysis. **Tuan-Anh Nguyen:** Writing – review & editing, Writing – original draft, Visualization, Validation, Investigation, Formal analysis, Data curation.

## Ethics approval and consent to participate

The University Medical Center Ho Chi Minh City ethics committees reviewed and approved this study protocol (reference number: 88/GCN-HĐĐĐ, signed on 29/09/2022). All procedures were conducted following relevant guidelines and regulations. Before the investigations, written informed consent was obtained from all participants and/or their legal guardians. This study adhered to the World Medical Association's Code of Ethics (Declaration of Helsinki) for human experiments.

## Consent for publication

Not applicable.

## Availability of data and materials

The detailed data and materials are available upon reasonable request (anh.nt@umc.edu.vn).

## Funding

The University Medical Center Ho Chi Minh City supported this work under Grant 92/2022/HĐ-ĐHYD. The funding agency had no role in the study's design, data collection, analysis, interpretation, or manuscript writing.

## Declaration of competing interest

The authors declare the following financial interests/personal relationships which may be considered as potential competing interests:The authors declare no conflict of interest, financial or otherwise.

## Data Availability

Data will be made available on request.
